# Anesthesia for intra-articular corticosteroid injections in juvenile idiopathic arthritis: A survey of pediatric rheumatologists

**DOI:** 10.1186/1546-0096-8-3

**Published:** 2010-01-13

**Authors:** Jennifer E Weiss, América G Uribe, Peter N Malleson, Yukiko Kimura

**Affiliations:** 1 Joseph M. Sanzari Children's Hospital, Hackensack University Medical Center, Hackensack, NJ 07601, USA; 2 British Columbia Children's Hospital, Vancouver, BC, Canada

## Abstract

**Objective:**

To determine the methods of anesthesia currently being used by pediatric rheumatologists when performing intra-articular corticosteroid injections (IACI).

**Study design:**

A questionnaire was emailed to all members of the Childhood Arthritis & Rheumatology Research Alliance, a pediatric rheumatology research network in North America. The questionnaire consisted of 11 questions ranging from procedure technique, treatments prescribed for topical anesthesia and oral analgesia, and factors that might affect procedural pain.

**Results:**

Seventy-four of 161 physicians (46%) responded to the questionnaire. On average, each physician injected 33 children (median 25, range 1-160) and 43 joints (median 30, range 1-150) yearly. Local anesthesia was used in children on average ≥ 8 years (range 2-16 years), with general anesthesia being more frequently used for younger children. All respondents used local anesthesia. The most commonly used methods of local anesthesia were EMLA^® ^cream plus subcutaneous lidocaine (58.8%), ethyl chloride spray only (39.7%), EMLA^® ^cream only (33.8%), subcutaneous lidocaine only (25%), and lidocaine iontophoresis only (11.8%). Buffering of the lidocaine was routinely done only 7.4% of the time.

**Conclusion:**

Although pediatric rheumatologists in North America perform IACI on a large number of patients each year, a wide variety of methods are used to deliver local anesthesia with no accepted standard of care. More studies are needed to determine the optimal method of local anesthesia delivery to minimize pain associated with IACI.

## Introduction

Intra-articular corticosteroid injections (IACI) are one of the mainstays of treatment for children with chronic arthritis [1-7]. IACI enables the physician to deliver localized treatment and may obviate the need for systemic medication or serve as an adjunct to systemic medications [1,2,4]. Another advantage is the rapidity of response to the IACI, which usually occurs within a few days of the injection [4]. IACI is most frequently used for children with oligoarticular juvenile idiopathic arthritis. If children have only one or two affected joints, it may be the only treatment used [1,3-5]. One of the disadvantages of IACI is the pain associated with the procedure. In one survey looking at the use of IACI for treatment of juvenile idiopathic arthritis, 43% of respondents regarded pain of procedure as a disadvantage to IACI therapy [7]. However, despite the frequent use of IACI by pediatric rheumatologists, there have been no studies performed to evaluate the relative efficacy of different methods of anesthesia, and consequently there seems to be no widely accepted standard of care for the provision of anesthesia in IACI.

Local anesthetics used for IACI may include EMLA^® ^cream [6], lidocaine iontophoresis [8] and subcutaneous lidocaine [4], as well as ethyl chloride spray or ELA-Max^® ^(4% liposomal lidocaine). EMLA^®^, a eutectic mixture of topical lidocaine 2.5% and prilocaine 2.5%, is a water emulsion cream base applied to skin under occlusion, either with a Tegaderm patch [3 M, St. Paul, MN] or plastic wrap 60 minutes prior to the procedure. It has been shown to decrease the pain in a number of minor procedures in children [9]. Although the only study formally assessing its role in IACI in children with chronic arthritis found it to be ineffective [6], it is commonly used by pediatric rheumatologists for IACI. Subcutaneous injection of lidocaine 2% solution is commonly used for many procedures including joint aspiration and injection [4]. The administration of lidocaine can be painful, but buffering it with sodium bicarbonate has been shown to make its injection significantly less painful [10].

Lidocaine iontophoresis is a safe, non-invasive way of delivering topical lidocaine. Low-level electrical current drives the lidocaine into the skin [11]. It is being used prior to IV cannulation [12], venipuncture [13], joint injections [8] and dermatologic procedures [14] with good results. The side-effects of iontophoresis include erythema, tingling and itching at the application site [11]. Rarely burns have been reported [11]. Iontophoresis may not be tolerated by all children due to the discomfort from the electrical current.

Some children, particularly very young children, or those undergoing injection of multiple joints, are sedated using such agents as propofol [4,6], midazolam [6,8], ketamine [4] and nitrous oxide [5]. The relative frequencies of use of different methods of local anesthetic and sedation by pediatric rheumatologists are unknown.

## Methods

Members of the Childhood Arthritis & Rheumatology Research Alliance (CARRA), a research network of all academic pediatric rheumatology centers in North America, received an 11 question self-administered, free response and multiple choice questionnaire between April and June 2005 either by email or distribution at the annual CARRA meeting inquiring about anesthesia for subjects receiving IACI. Questions pertained to procedure technique, medications prescribed for topical anesthesia, oral analgesia, the use of sedation, and factors affecting their use. The responding physician's beliefs regarding their patients' experience of pain during the procedure were examined, as were the important factors affecting the level of procedural pain as per the responding physician.

## Results

Seventy-four of 161 physicians (46%) (55 from the USA and 15 from Canada) responded to the questionnaire. Respondents reported injecting a mean of 33 children (median 25, range 1-160) and 43 joints (median 30, range 1-150) yearly. Forty-three percent of physicians reported that they aspirate the joint to "dryness" prior to injecting corticosteroids. Local anesthesia was reported to be used in children with a median age of 8 years (2-16 years). Although a wide variety of local anesthetic methods were used (see Table 1), all respondents use local anesthesia for IACI. Factors affecting the physician's decision to use local or general anesthesia are listed in Table 2.

**Table 1:** Local anesthesia delivery methods* used by pediatric rheumatologists for intra-articular corticosteroid injections

Method	Percent
EMLA†^® ^cream + subcutaneous lidocaine	58.8
Ethyl chloride spray	41.2
EMLA^® ^cream	33.8
Subcutaneous lidocaine	
Non-buffered lidocaine	29.4
Routine buffering of lidocaine	7.4
Lidocaine iontophoresis	11.8

* Use of more than one local anesthesia method for IACI was reported by 60% of responding physicians; † Eutectic mixture of topical lidocaine.

**Table 2:** Factors determining the type and method of anesthesia use during intra-articular corticosteroid injections

Factor	Percent
Patient age	79.4
Number of joints to be injected	75.0
Parental anxiety	45.6
Patient anxiety	82.4
Joint size	33.8
Availability of insurance	4.4

Conscious sedation or general anesthesia was used by 85% of physicians. Sixty percent reported use of more than 1 sedative. At many institutions, the agent used was at the discretion of the anesthesiologist. Conscious sedation or general anesthesia was used for the following reasons: patients ≤ 8 years of age, patients needing multiple joints injected and patients having IACI of small joints such as in the hand. The most frequently used agents for conscious sedation are listed in Table 3.

**Table 3:** Frequency and agents used for sedation during intra-articular corticosteroid injections

Method	Percent
Midazolam (IV)	39.5
Midazolam (oral)	9.0
Propofol	19.0
Fentanyl or vistanyl	19.0
Ketamine	12.0
Gas (sevoflurane, nitrous oxide, isoflurane, halothane)	30.0
Other	16.0

* Use of more than one sedative for IACI was reported by 60% of responding physicians.

Physicians reported that they believe the majority of their patients (78%) experience mild or no pain during IACI, with only 4% of patients experiencing severe pain. Sixty-seven percent of physicians believe that giving unbuffered subcutaneous lidocaine may actually increase procedural pain, but only 7% of physicians buffered the lidocaine. Other factors cited as potentially contributing to procedural pain included: patient (57%) and parent (68%) anxiety regarding the procedure, multiple joint injections at one time (38%), young patient age (57%), injection of small joints (10%), severity of the arthritis (7%), difficulty of aspiration/injection (4%), history of prior joint injection without the use of anesthesia (3%) and physician confidence and speed (3%). Forty-four percent of physicians prescribed oral analgesics routinely for use after the IACI. The type of analgesic used was not assessed.

## Discussion

Although pediatric rheumatologists in North America perform IACI on a large number of patients each year, a wide variety of methods are used to provide anesthesia, and there is no accepted standard of care regarding the best methods to be used. Although most patients were thought to experience no pain or mild pain using various methods of local anesthesia, it is known that nurses and physicians often underestimate procedural pain [5].

Many physicians were concerned that unbuffered subcutaneous lidocaine contributed significantly to the pain of the procedure, but very few attempted to prevent this pain by buffering the lidocaine. It is not known if physicians are unaware of the benefits of buffering the lidocaine or if it is impractical in the physician's office.

It is interesting that less than half of the respondents aspirated the joint prior to injection, since Weitoft, et al found that synovial fluid aspiration reduces the risk of relapse in rheumatoid arthritis patients [15]. There are a number of reasons that may make it more difficult to aspirate the joint prior to IACI in the pediatric patient, such as added discomfort (especially if attempting to aspirate the joint to dryness) and difficulty holding still (if local anesthesia is being used). A similar study in pediatrics would be useful to address the benefit of fluid aspiration prior to IACI.

Limitations of this study include responder bias and physician self-reporting. Little is known about the child or the parent's perception of pain during IACI using the different methods of anesthesia, and this study was not able to address this issue.

Given the variety of methods used by pediatric rheumatologists, it is probable that there is a significant variation in the actual amount of procedural pain being experienced by children with JIA undergoing IACI. More studies are needed to address this issue and to determine the optimal methods of providing pain relief delivery during this commonly used procedure.

## Competing interests

The authors declare that they have no competing interests.

## Authors' contributions

JW contributed to the conception, design and coordination of the study and drafted the manuscript. YK contributed to the conception, design and coordination of the study. PM contributed to the conception and design of the study. AU contributed to the conception and design of the study and performed statistical analysis. All authors read and approved the final manuscript.
